# Dynamic control of the directional scattering of single Mie particle by laser induced metal insulator transitions

**DOI:** 10.1515/nanoph-2024-0154

**Published:** 2024-07-01

**Authors:** Yanlin Zhu, Shulei Li, Yang Zhang, Jinjing Meng, Xu Tan, Jingdong Chen, Mingcheng Panmai, Jin Xiang

**Affiliations:** Key Laboratory of Optoelectronic Technology & Systems, Ministry of Education, and College of Optoelectronic Engineering, 47913 Chongqing University, Chongqing 400044, China; School of Optoelectronic Engineering, Guangdong Polytechnic Normal University, Guangzhou 510665, China; College of Physics and Information Engineering, Minnan Normal University, Zhangzhou 363000, China; School of Electrical and Electronic Engineering, Nanyang Technological University, Singapore 639798, Singapore

**Keywords:** vanadium dioxide, Mie resonance, insulator-metal transition, all-optical modulator

## Abstract

Interference between the electric and magnetic dipole-induced in Mie nanostructures has been widely demonstrated to tailor the scattering field, which was commonly used in optical nano-antennas, filters, and routers. The dynamic control of scattering fields based on dielectric nanostructures is interesting for fundamental research and important for practical applications. Here, it is shown theoretically that the amplitude of the electric and magnetic dipoles induced in a vanadium dioxide nanosphere can be manipulated by using laser-induced metal-insulator transitions, and it is experimentally demonstrated that the directional scattering can be controlled by simply varying the irradiances of the excitation laser. As a straightforward application, we demonstrate a high-performance optical modulator in the visible band with high modulation depth, fast modulation speed, and high reproducibility arising from a backscattering setup with the quasi-first Kerker condition. Our method indicates the potential applications in developing nanoscale optical antennas and optical modulation devices.

## 1 Introduction

Subwavelength dielectric nanoparticles exhibit multi-order Mie resonance, i.e., electric dipole (ED) and magnetic dipole (MD), electric quadrupole (EQ), and quadrupole (MQ), etc., which can trap energy in space and time domain, thus have received intensive and extensive studies in recent years [1], [2], [3], [4], [5]. It is widely utilized in the fields of enhanced light–matter interaction [6], [7], [8], nonlinear optics [9], [10], nano-optical antennas [11], [12], [13], and optical sensing [14], [15], [16]. Since the Mie resonance of dielectric nanoparticles can be controlled by optimizing the geometric parameters, the scattered field can be tailored through the interference between the electric and magnetic dipoles supported by nanoparticles [5], [12], [17], [18]. When the electric and magnetic dipoles perfectly constructively or destructively interfere, it leads to pure backward and forward scattering (BS/FS), known as the first and second Kerker conditions, respectively [4], [19], [20]. In addition, it has then been extended to other shapes of Mie nanostructure to manipulate scattering not only at the forward and backward directions but also other possible scattering angles be referred to as the generalized Kerker effect [21], [22], [23], such as transverse scattering induced by interference between high order Mie resonance [24], [25], [26], [27], which is widely utilized in high-efficiency directionally radiating optical nano-antennas.

Nanoscale all-optical modulators can control and manipulate light on the optical wavelength length scale, which is widely utilized in communications, sensing, and optical data storage. So far, all-optical modulators are mainly based on the electro-optic effect [28], [29], [30], thermo-optic effect [31], [32], [33], optical nonlinear Kerker effect [19], [34], [35], [36], [37], carrier injection [38], [39], [40] and so on. Despite the above methods having been widely utilized in industry and studied in academic research, a bottleneck issue, only a little change of dielectric constant can be realized. Phase-change materials (PCMs) can drastically change the refractive index by changing the lattice of the substance under an external heating excitation, which has been widely utilized in the field of photonic devices such as optical data storage [41] and optical switching [42]. Among them, Ge_2_Sb_2_Te_5_ (GST) has the ability to realize a dramatic optical contrast (*δ*_*n*_ > 2) between these two states in response to crystallization or amorphization processes. Strong and omnidirectional light absorption from ultraviolet to near-infrared using GST metasurface. Active control of anapole states by structuring the phase-change alloy GST. Dynamic thermal emission control based on ultrathin plasmonic metamaterials, including phase-changing material GST. Also, vanadium dioxide (VO_2_) exhibits a reversible insulator-metal transition (IMT) at near room temperature (*T*_*c*_ = 68 °C) with a large refractive index contrast and fast switching time, which has attracted great interest in recent years in the field of optical modulators [32], [43], [44]. Despite great success in building VO_2_-based optical modulation devices with various functionalities, there are limitations that need to be improved. First, current VO_2_-based optical modulators are mainly based on the heating platform experimental setup, resulting in slow modulation speeds. Second, the refractive index constant between the insulator phase and the metal phase of VO_2_ is only noticeably manifested in the infrared band [31], [45], [46], resulting in the difficult implementation of optical modulators in the visible band. It has been shown that VO_2_ reproducible optical modulators demonstrated using the photothermal effect of a pulsed laser [47]. Owing to plasmons antenna-assisted nanoscale VO_2_ excitation at short-wave infrared, it has 20 times reduced switching energies and 5 times faster recovery times than a VO_2_ film without antennas [47]. In addition, combining VO_2_ with localized plasmon resonance of Au nanoparticles demonstrated an optical modulator in the visible region with modulation depths of ∼31 % in the 630 nm [48]. Although considerable efforts have been made, the nanoscale optical modulators in visible bands with high modulation depths are still quite challenging.

In this article, we have theoretically proposed and experimentally demonstrated the dynamic control of the directional scattering of a single Mie sphere with photo-thermal induced metal-insulator transitions. As a proof-of-principle application, we have demonstrated a high modulation depth and high reproducibility optical modulator in the visible band using a backscattering setup with the quasi-first Kerker condition.

## 2 Results and discussion

**Figure 1:**
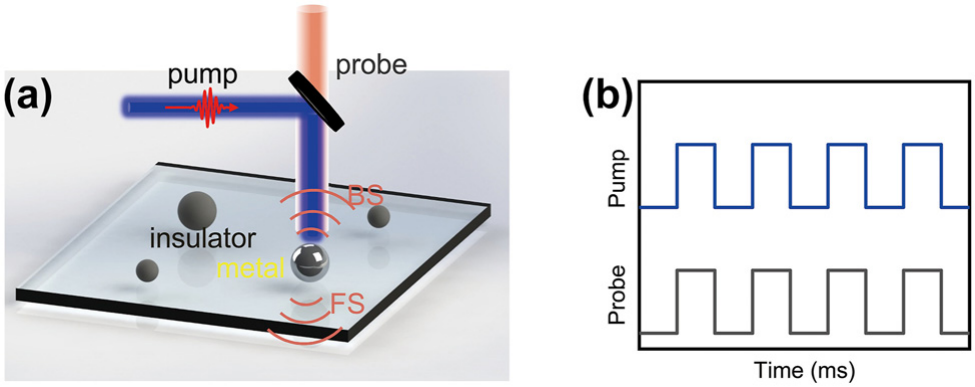
Schematic of the optical VO_2_ NPs modulator. (a) Experimental schematic of dynamic control of directional scattering of single VO_2_ NPs by the laser induced IMT. (b) Schematic comparison of phase transition modulation schemes is that the modulated successive optical pump pulses (blue) acts on the particle in conjunction with the probe light (black).

Figure 1(a) shows the schematic of an all-optical VO_2_ modulator exciting with a 488 nm laser and probing the scattering light with a broadband white light source in the forward/backward direction. Insulating state VO_2_ nanoparticles (NPs) fabricated by using fs laser ablation [9] were randomly placed on the quartz slide at room temperature, which can transition metal using 488 nm laser-induced photothermal *in situ* with a reversible process, as shown in Figure 1(b). The quality of VO_2_ NP can be improved by using femtosecond laser ablation with a short focal length objective [49]. Also, the state-of-the-art nanofabrication techniques using electron-beam lithography combined with dry etching can improve the morphological diversity of the VO_2_ nanostructure [50]. Unlike phase transition performed by a heating platform, the photo-thermal induced phase transition has a femtosecond response time and can be achieved by *in situ* localized heating [47], [51], [52].

The refractive index of the VO_2_ crystal in insulating and metallic state Ref. [53]. In the insulating state, the refractive index real part *n* can be up to 3 with a negligible imaginary part *k*, resulting in a notable Mie resonance in the visible. In contrast, since the presence of a high density of electrons in the metallic state VO_2_, there is a significant absorption with a sizeable imaginary part *k* of the refractive index, as shown in Figure S1 (see Supplementary Material, Figure S1). Note that the discrepancies of the refractive index between the metallic and insulating phases are obscure in the visible region, implying that it is difficult to realize a VO_2_-based optical modulator in the visible [54].

**Figure 2:**
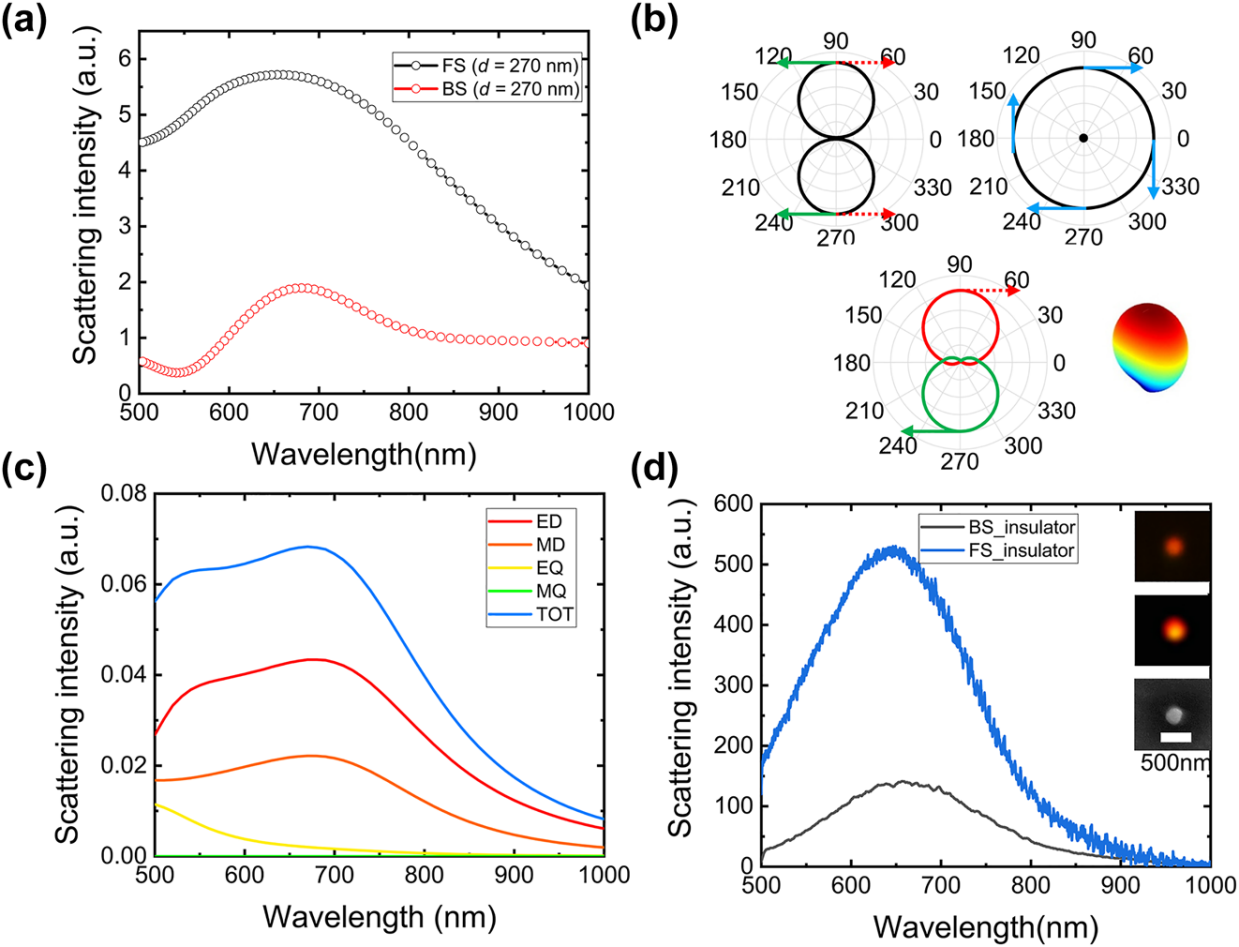
Directional scattering spectra of insulating phase VO_2_ NPs. (a) Simulated FS (black) and BS (red) spectra of VO_2_ NP for radius *d* = 270 nm. (b) Schematic showing directional radiation patterns induced by the interference between of MD and ED. The inset showing the 3D radiation pattern at quasi first Kerker condition 640 nm. (c) Decomposition of the total scattering spectra simulated for an insulating phase VO_2_ NP with *d* = 270 nm. (d) Measured scattering spectra of VO_2_ NP with radius *d* ∼ 270 nm at BS (black) and FS (blue). The FS imaging, backs cattering imaging recorded by using a charge-coupled device (CCD) and SEM image of the VO_2_ NP are shown in the insets. The length of the scale bar is 500 nm.

Figure 2(a) shows the FS and BS spectrum of VO_2_ NP with a diameter *d* = 270 nm for the insulating state. One can see that the far-field radiated power density dominated by FS. Also, we have simulated the evolution of the full-field/forward/backward scattering spectrum with increasing diameter (*d*) calculated for VO_2_ NPs in insulating state, as shown in Figure S2 (see Supplementary Material, Figure S2). One resonance peak is observed for the FS spectrum, which redshifts gently with increasing diameter. In contrast, the peak of the backscattering spectrum rushes from 500 to 800 nm, following a high-order resonance peak at a large diameter. Since the coherence effects between electric and magnetic dipoles, one can tailor the far-field scattering of VO_2_ NPs. Figure 2(b) shows that electric and magnetic dipoles result in zero-BS, which is called the first Kerker condition. Such sources are interesting as elements of small antennas to realize ‘Huygens’ metasurface [19], [55]. Correspondingly, anti-phase electric and magnetic dipoles induce pure BS, which is called the second Kerker condition.

To gain deeper insight into a link between dipole moments of insulating and metallic state, we decomposed the total scattering spectra of a VO_2_ NP with *d* = 270 nm by multipole expansion theory in the Cartesian coordinate system [56], [57], as shown in Figure 2(c). In this case, the multipole moments induced in the VO_2_ NPs can be calculated by integrating the induced polarization currents over the volume of the VO_2_ NP, including the multipoles: electric dipole (ED), magnetic dipole (MD), electric quadrupole (EQ), magnetic quadrupole (MQ), and toroidal dipole (TD). The calculations are expressed as follows: ED: **p** = *∫* **P**d**r**. MD: $m=-\frac{i\omega }{2}\int r\times Pdr$ . EQ: $eq=3\int \left(rP\left(r\right)+P\left(r\right)r-\frac{2}{3}\left[rP\left(r\right)\hat{U}\right]\right)dr$ . MQ: $mq=\frac{\omega }{3i}\int \left(\left[r\times P\left(r\right)\right]r+r\left[r\times P\left(r\right)\right]\right)dr$ . Here, The polarization **P** induced in the VO_2_ NPs can be calculated based on the electric field **E**(**r**). **P** = *ɛ*_0_(*ɛ*_*p*_ − *ɛ*_*d*_)**E**, *ɛ*_0_, *ɛ*_*p*_, and *ɛ*_*d*_ are the permeabilities of vacuum, nanoparticle (VO_2_ in our case) permeabilities of vacuum and environment (air in our case). We obtain the 3D electric field of VO_2_ nanoparticles at the desired wavelengths using finite element method simulations. Given the known values of *ɛ*_0_, *ɛ*_*p*_, and *ɛ*_*d*_, we can determine the polarization **P**. Subsequently, all the multipolar moments can be derived using the above formula. One can observe that although the electric and magnetic dipole are in phase, their amplitudes are different with a ratio ∼50 %, implying that the scattering field of VO_2_ NP is dominated by FS with a partial BS, as shown in Figure 2(a). Figure 2(d) shows the experimentally measured FS and BS spectra of VO_2_ NP with a diameter *d* ∼ 270 nm at the insulator state. The scanning electron microscope and optical microscope characterization of the VO_2_ NPs randomly placed on the quartz slide was shown in Figure S3 (see Supplementary Material, S3). The VO_2_ NPs fabricated by femtosecond laser ablation are not perfectly spherical in actual experiments. Here, we have performed a simulation to calculate the scattering spectrum of the elliptical VO_2_ NPs. (See Supplementary Material, S4) One can see that the FS and BS of VO_2_ NP experimentally measured has a resonance peak near 700 nm, agreeing well with the simulation. Note that since FS and BS do not have the same measurement optical setup, the absolute intensities of Figure 2(d) are not meaningful information.

**Figure 3:**
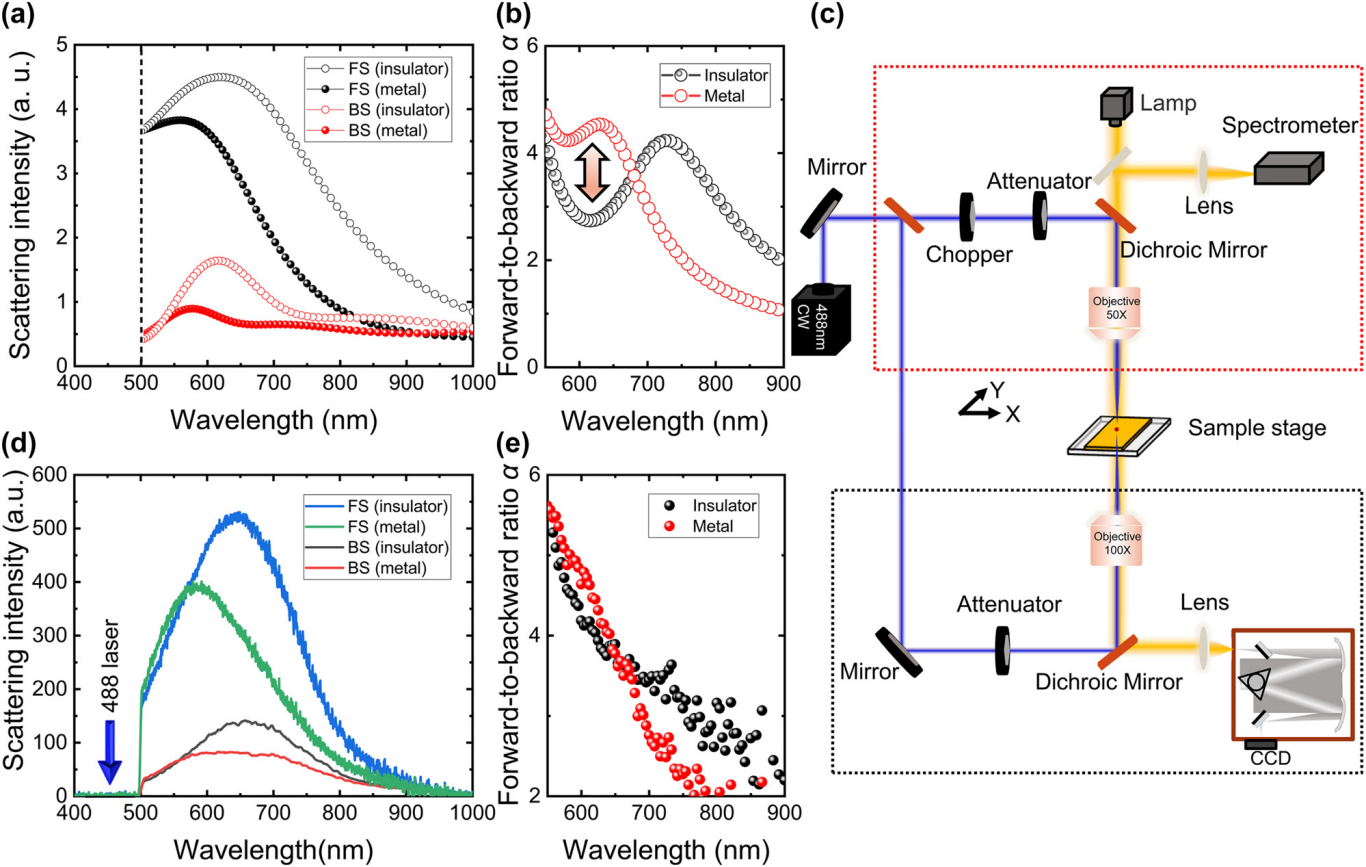
Experimental demonstration of the dynamic control directional scattering of single VO_2_ sphere by laser induced metal insulator transitions. (a) Simulated FS (black) and BS (red) spectra of VO_2_ NP for radius *d* = 270 nm. (b) The evolution of the forward-to-backward ratio with increasing wavelength for insulator (balck) and metallic (red) phases of VO_2_ NP. (c) Experimental setup of dynamic control of directional scattering of single VO_2_ sphere, where the red/black dashed boxes represent the BS/FS light paths, respectively. (d) Measured scattering spectra of VO_2_ NPs with radius *d* = 270 nm for FS (insulating phase is black and metallic phase is red) and BS (insulating phase is blue and metallic phase is green). (e) Experiment measured the evolution of the forward-to-backward ratio with the wavelength for insulator(balck) and metallic(red) phases of VO_2_ NPs.

The multipole moments of the VO_2_ NP with the metallic state are shown in Figure S5 (see Supplementary Material, S5). Comparing the multipole moments of the insulator VO_2_, it can be seen that the ratio of ED and MD amplitudes for the metallic state can be up to 70 %, resulting in a significantly reduced BS at the quasi-first Kerker’s wavelength ∼640 nm. Figure 3(a) shows simulated FS and BS spectra of VO_2_ NP for the insulator and metallic state. After the IMT, the BS of VO_2_ NP decreased by 50 % at the resonance peak ∼640 nm, while FS only reduced by approximately 20 %, resulting in a drastic increase in the forward-to-backward ratio. We have calculated the evolution of the forward-to-backward ratio with increasing wavelength for the insulator and metallic phases of VO_2_ NP, as shown in Figure 3(b). One can observe that the backward-to-forward scattering ratio changes from 2.4 to 4.5 when the VO_2_ NP transit into a metallic form insulator state, implying the directional scattering of resonant VO_2_ NP modulated by repeatable IMT process.

To experimentally demonstrate this modulation effect with a rapid phase transition time, we employ a laser-induced photothermal effect to realize the IMT of VO_2_, as shown in Figure 3(c). In experiments, we excited the VO_2_ NPs by using a 488 nm continuous wave (CW) laser with a broadband probe white light. A mechanical chopper was used to demonstrate reversible phase change by controlling the number and duration of the pump laser. A 50× objective lens was used to focus the pump and probe beam onto the VO_2_ NP. A grating spectrometer (SR-500, Andor) and fiber spectrometer (QE pro, Ocean Insight) was utilized to measure the FS and BS spectra of the VO_2_ NPs, respectively. Since VO_2_ has a significant absorption in the violet light band, the 488 nm laser can rapidly increase the temperature of the nanoparticles, leading to an IMT, as shown in Figure 3(d). Here, the VO_2_ NP performed in the experiment is the same as in Figure 2(b), with a good agreement between the calculated and measured results. The dark field spectra of VO_2_ NPs with different diameters are shown in Figure S6 (see Supplementary Material, S6). Also, we have calculated the evolution of the forward-to-backward ratio for experimental data insulating and metallic state of VO_2_ NP based on Figure 3(d), as shown in Figure 3(e), which clearly shows that the forward-to-backward ratio of the VO_2_ NP can be manipulated using laser-induced IMT.

**Figure 4:**
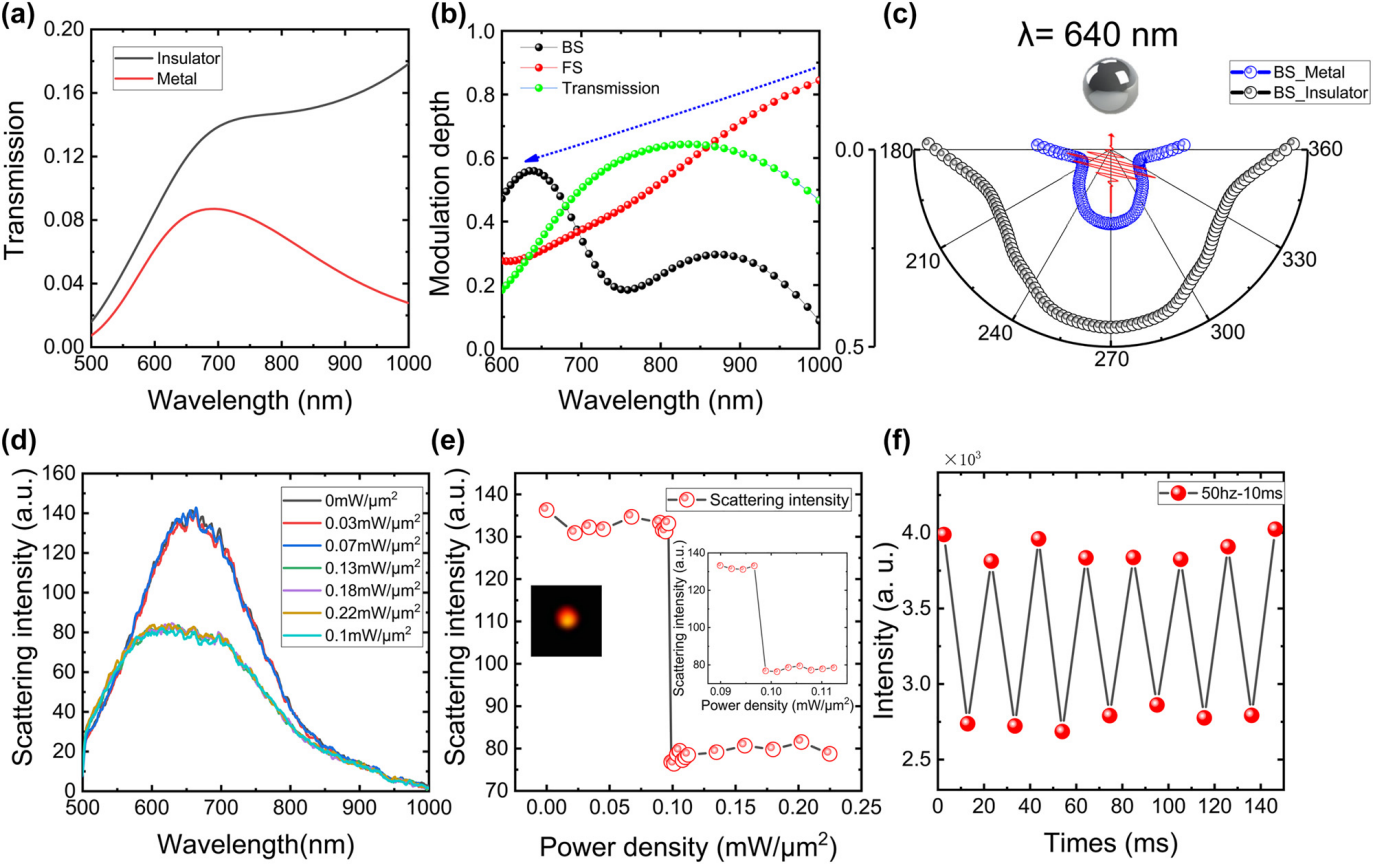
Experimental demonstration the high performance visible optical VO_2_ NPs modulator based on quasi-first Kerker condition. (a) The transmittance of 240 nm VO_2_ film for insulator (balck) and metallic (red) phases. (b) Comparison of modulation depths for FS/BS of radius *d* = 240 nm NP and transmission of 240 nm films. (c) The BS radiation patterns of VO_2_ NP with a radius *d* = 240 nm calculated at wavelengths of *λ* = 640 nm. (d) Evolution of the experimentally measured BS spectrum of VO_2_ NP with different pump laser intensity. (e) The dependence of intensity at the peak (∼640 nm) of the BS spectrum on the laser power (0–0.22 mW/μm^2^). Evolution of the BS spectral peak intensity with the critical laser power density (0.09–0.11 mW/μm^2^) is shown in the insets. (f) BS Spectral intensity variation of IMT process for VO_2_ NP under 50 Hz modulation frequency with a fixed laser power density ∼0.22 mW/μm^2^.

Furthermore, we evaluated the performance of several kinds of VO_2_-based optical modulator scenarios in detail, including the transmittance of VO_2_ film, the FS and BS spectra of the VO_2_ NP. Figure 4(a) shows the evolution of the transmittance with the phase transition for the VO_2_ film with a thickness of 240 nm, showing an obvious difference in the short-wave infrared and neglectable in the visible band. In order to quantitatively analyze the modulation of different schemes, we define the modulation depth *δ* = 1 − *T*_*m*_/*T*_*i*_, where *T*_*m*_ and *T*_*i*_ represent the response spectra of the metallic and insulating state, respectively. As previously predicted roughly using the refractive index contrast of the IMT, the modulation effect of the pure VO_2_ film exists mainly in the near-infrared region. Interestingly, the interaction of the electric and magnetic dipoles of VO_2_ NP induces directional scattering at the quasi-first Kerker’s wavelength, enabling a high-contrast spectral modulation to the visible band. Figure 4(b) shows that the FS and BS scenarios realized a high contrast modulation at 750 nm and 640 nm, with a modulation depth of 65 % and 57 %, as shown in the green and black curves, respectively. In Figure 4(c), we show the *x*–*z* plane BS radiation patterns of the VO_2_ NP calculated for the metallic and insulating phases for a wavelength ∼640 nm. It can be seen that the BS of the VO_2_ NP is radiation with different intensities, depending strongly on the state of the VO_2_ NP. The *x*–*z* plane FS patterns of the VO_2_ NP with wavelength ∼750 nm were calculated for the metallic and insulating phases, as shown in Figure S7 (see Supplementary Material, S7).

To experimentally explore the phase transition critical temperature of the VO_2_ NP, we have performed experiments to measure the evolution of the BS spectrum with increasing pump laser intensity, as shown in Figure 4(d). When the incident laser power density exceeds 0.1 mW/μm^2^, the BS intensity of VO_2_ NP significantly decreases, indicating there is a phase transition. The dependence of intensity at the peak (∼640 nm) of the BS spectrum on the laser power density is shown in Figure 4(e). Detailed data for the intensity of pump lasers at 0.09–0.11 mW/μm^2^ are shown in the inset. Note that in addition to the strong absorption of VO_2_ in the violet laser, the ultralow phase transition threshold power density (0.099 mW/μm^2^) is also attributed to the Mie resonance of VO_2_ NP confine electromagnetic field to subwavelength scales, enhancing the laser-induced photothermal effect.

Since VO_2_ can be modulated at picosecond speeds, we reveal the phase transition speed of VO_2_ NP by simply using a chopper with repetition rates *f* = 50 Hz to switch the pump laser beam. Here, the intensity of the pump laser was fixed at 0.22 mW/μm^2^. Figure 4(f) demonstrates the all-optical switching realized by utilizing the ultralow threshold phase transition of VO_2_ NP. For comparison, we also have performed experiments to show how the scattering spectrum of the VO_2_ NPs can be modulated by the heating platform without lasers(See Supplementary Material, S8). Compared to the thermally induced phase transition, the photo-induced phase transition strategy is compact with a rapid switching speed. More importantly, we can control the phase transition of the Mie sphere at the nanoscale without affecting other devices, which gives rise to broad applications in compact optical switching in the visible band, confirmed by the electromagnetic field distribution of the VO_2_ NP (see Supplementary Material, 9).

**Figure 5:**
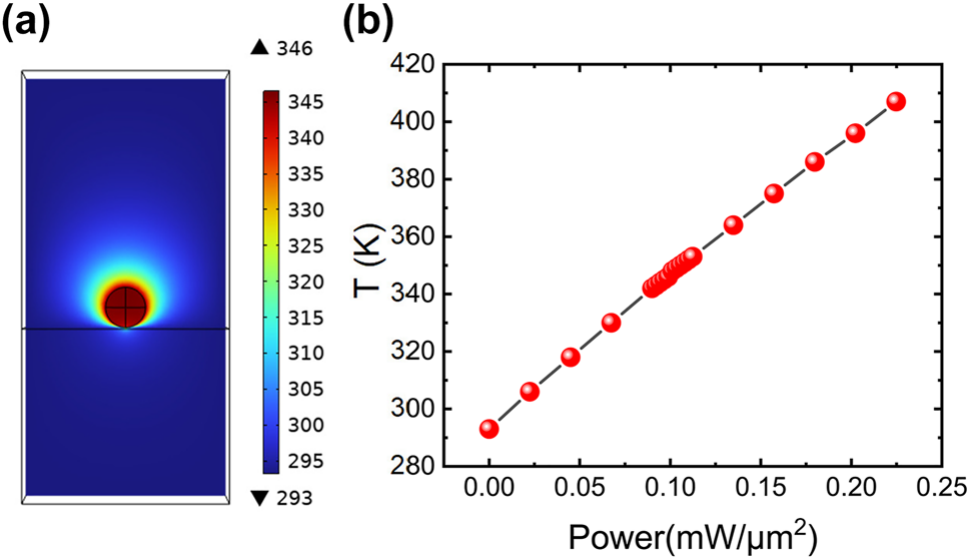
Simulated laser-induced photothermal effect of VO_2_ NPs. (a) Static temperature distribution in the *XZ* plane calculated for a VO_2_ NP excited by using a 488 nm CW laser with a power density 0.099 mW/μm^2^. (b) The dependence of laser-induced photothermal temperature of VO_2_ NP on pump laser power density.

The temperature changes induced by continuously pumped laser light in nanoparticles were calculated numerically based on the finite element method (COMSOL Multiphysics 6.0). We calculated the temperature inside the nanoparticles at different pumping power densities based on the Beer–Lambert law:

(1) $$\rho C_{p}\frac{\partial T}{\partial t}-\nabla \cdot \left(k\nabla T\right)=Q$$

where, *ρ* = 4,340 kg/m^3^ and *C*_*p*_ = 690 J/(kg K) are the material density constant and pressure heat capacity [58], respectively. We have calculated the temperature of VO_2_ NPs by combining the electromagnetic waves, frequency domain (ewfd) module with heat transfer in solid (ht) module. The VO_2_ NP with *d* = 270 nm on a quartz substrate was exposed to 488 nm electromagnetic waves with *x*-polarized. We use perfectly matched layer (PML) boundary conditions to eliminate the reflected electromagnetic waves by the boundary. Non-uniform grids with the minimum size of 2 nm were used. The heat source term *Q* is equal to the absorbed light related to the incident light electromagnetic power density loss density. In Figure 5(a), we show the static temperature distribution achieved in VO_2_ NP under continuous wave laser excitation with a power density 0.099 mW/μm^2^. The temperature distribution inside the nanoparticle is almost uniform due to the nanoscale volume. Theoretically, the phase transition temperature of VO_2_ is around 341 K. In the actual experiment, the 0.099 mW/μm^2^ laser of our phase transition reaction corresponds to the internal temperature of the nanoparticles of 346 K, which satisfies the conditions for the phase transition.

**Figure 6:**
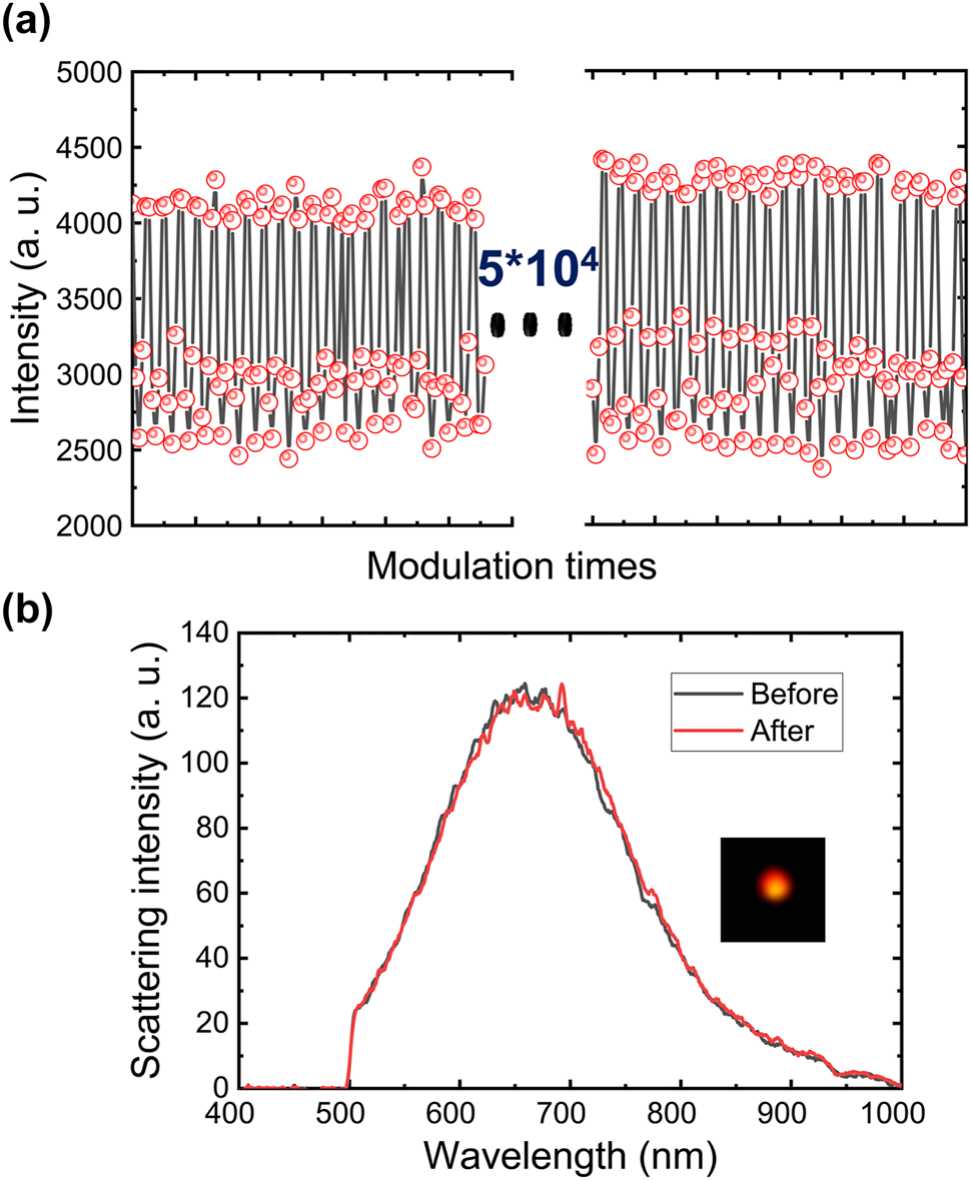
Experiment characterization the repeatability performance of the VO_2_ NP based modulator. (a) BS Spectral intensity variation of the IMT process of VO_2_ particles at 20 Hz for 5 × 10^4^ times modulations with 0.22 mW/μm^2^ laser power density. (b) BS spectra before (black) and after (red) 5 × 10^4^ times laser modulations.

As a key parameter of optical switching, repeatability represents the reversibly switched times while the device has not been broken [59]. Figure 6(a) presents the repeatability of VO_2_ NP utilizing overlapping excitation of the pump laser with repetition rates of 20 Hz. It is easy to repeat 5 × 10^4^ times optical modulate in a visible band using a pump laser mild intensity 0.22 mW/μm^2^, with a high modulation depth(∼50 %). To confirm the quality of VO_2_ NP after the 5 × 10^4^ times optical laser pulse excites, we measured the BS spectra of VO_2_ NP, as shown in Figure 6(b). As we can see, the dark field scattering of VO_2_ NP did not any change after tens of thousands of laser pulse modulations, implying that VO_2_ NP was not destroyed by the laser. In order to highlight the improvement achieved recently with different types of VO_2_-based optical modulators, we compare the experimental performance as shown in Table 1 (see Supplementary Material, Table 1)

## 3 Conclusions

In conclusion, we propose and experimentally demonstrate a scheme for dynamic control of directional scattering for a single Mie sphere by utilizing laser-induced metal-insulator transitions. By controlling the strengths of the electric and magnetic dipoles of VO_2_ Mie sphere under the quasi first Kerker effect condition, we can substantially increase the modulation depth in the visible band. In particular, we experimentally demonstrate using BS as a probe signal with a modulation depth of up to 57 % at 640 nm, 2.2 times that of a pure VO_2_ film. After 5 × 10^4^ times switching experiments, the VO_2_ Mie sphere still maintains high performance. Owing to the Mie resonance of VO_2_ NPs electromagnetic field localization effect, the threshold power density of the VO_2_ modulator is only ∼0.1 mW/μm^2^. The method proposed here offers insights for designing visible optical modulators and developing reconfigurable optics for nanoscale optical devices.

## Supplementary Material

Supplementary Material Details
