# Supplementary material for: Dynamic control of the directional scattering of single Mie particle by laser induced metal insulator transitions
Source: Nanophotonics. 2024 Jul 1;13(20):3815–23. doi: 10.1515/nanoph-2024-0154 (PMC11466016; doi:10.1515/nanoph-2024-0154)
Supplement: Supplementary file 1 — Supplementary Material Details [file j_nanoph-2024-0154_suppl_001.pdf]

Supplementary Material

Dynamic control of the directional scattering of Mie particles using laser induced metal insulator transitions

YanLin Zhu, Shulei Li, Yang Zhang, JinJing Meng, Xu Tan, Jingdong Chen, Mingcheng Panmai, and Jin Xiang*

YanLin Zhu, Yang Zhang, JinJing Meng, Xu Tan, Jin Xiang, Key Laboratory of Optoelectronic Technology & Systems, Ministry of Education, and College of Optoelectronic Engineering, Chongqing University, Chongqing 400044, email:China,jin@cqu.edu.cn

Shulei Li, School of Optoelectronic Engineering, Guangdong Polytechnic Normal University, Guangzhou 510665, China

Jingdong Chen, College of Physics and Information Engineering, Minnan Normal University, Zhangzhou 363000, China

Mingcheng Panmai, School of Electrical and Electronic Engineering, Nanyang Technological University, Singapore 639798,Singapore

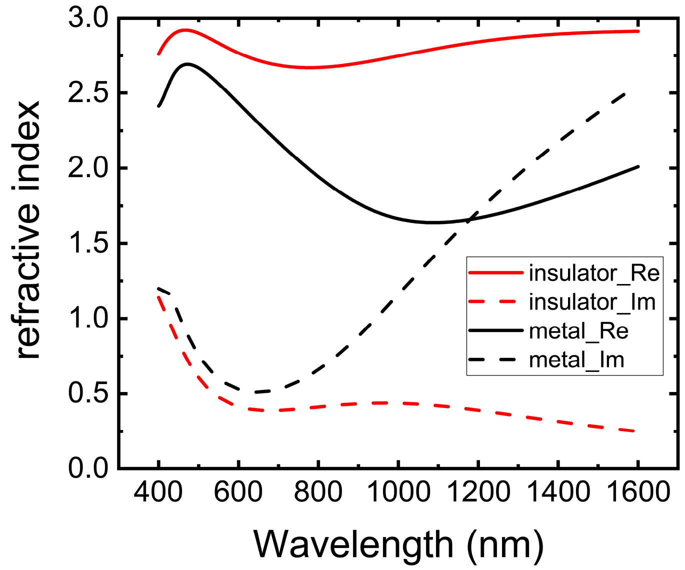

Figure S1： The real and imaginary refractive index of a VO_2 crystal in its insulating and metallic state.

In practical experiments, the scattering properties of nanoparticles, only FS and BS can be analyzed. Through simulations via CMOSOL, we've tracked how these scattering spectrums evolve as VO_2 nanoparticles' diameter (*d*) increases from 180 to 300 nm. Our findings, detailed in Figure S2, demonstrate the spectrum's evolution in both metallic and insulating phases, highlighting a pronounced FS intensity at resonant wavelengths compared to BS, which is a result of the coherent interaction between ED and MD modes.

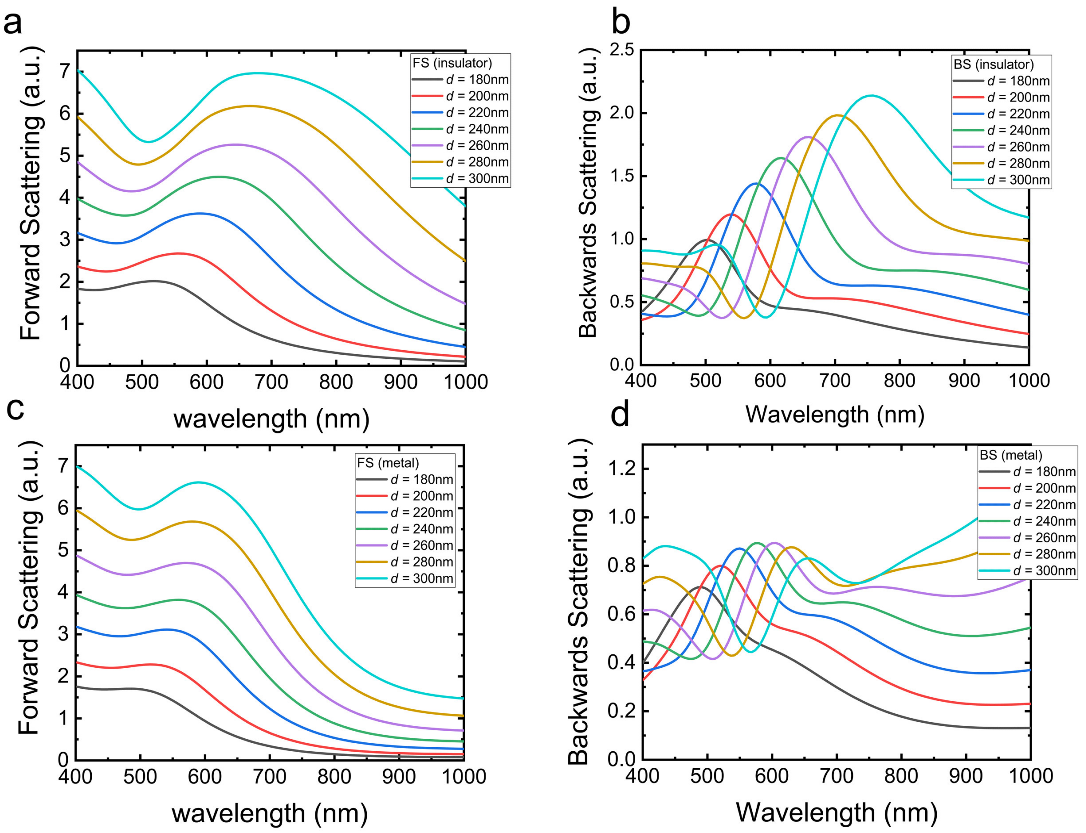

Figure S2: Evolution of the FS and BS spectrum with increasing diameter (*d*) calculated for a VO_2 NPs with insulating(a,b) and metallic(c,d) state, respectively.

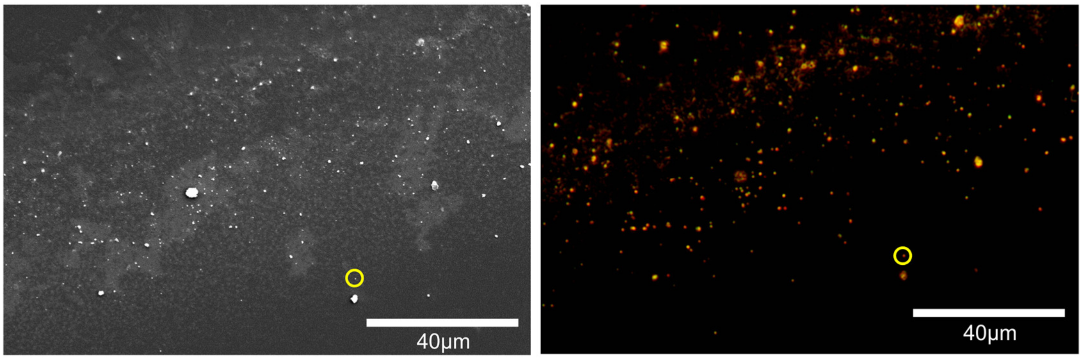

Figure S3: Scanning electron microscope (a) and optical microscope(b) characterization of the VO_2 NPs randomly placed on the quartz slide.

Since the VO2 NPs fabricated by femtosecond laser ablation are not perfectly spherical in actual experiments, we have performed simulations to calculate the scattering spectrum of the elliptical VO_2 NPs. In the simulation, we define the three diameters of the ellipsoid as d_x, d_y, and d_z, respectively. The d_z = 240nm was set as a constant. While increasing d_x, we decrease d_y (with d_x ranging from 180-240nm and d_y ranging from 300nm-240nm) to maintain the volume of the NPs within a constant, as illustrated in Figure S4(a) inset. The incident beam propagates along the z-axis and the electric field vector is along the *x* axis, i.e., *x*-polarization. The simulation shows that the

peak of scattering spectrum of VO_2 NPs are blueshift as the d_x decreases, as shown in Figure S4(b)-(d).

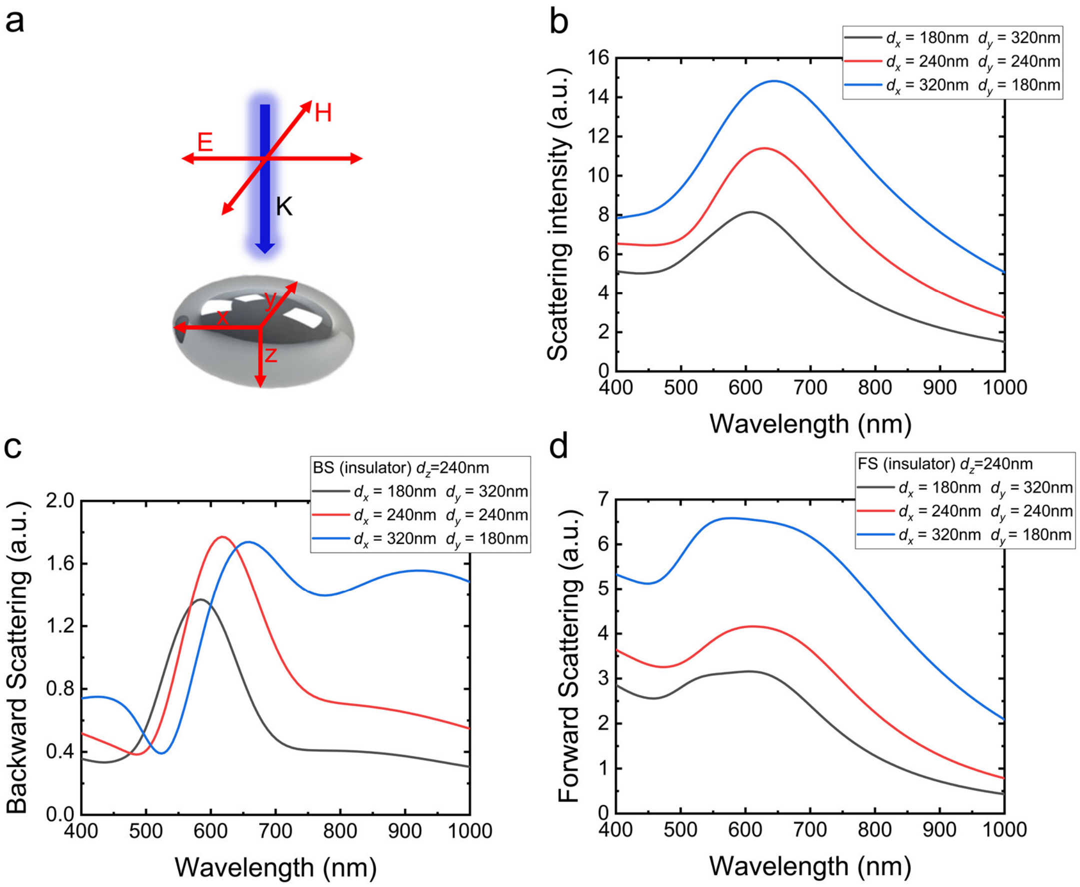

Figure S4. (a) Schematic of the setup of simulation. (b-d) Evolution of the full-field scattering (b), FS(c) and BS(d) spectrum with increasing diameter d calculated for ellipsoidal VO_2 NPs with insulating state, respectively.

The multipole moments of the VO_2 NP with the metallic state. To investigate the directional scattering differences of VO_2 NP between insulating and metallic states, we employed multipole expansion theory to dissect the total scattering spectrum of a metallic VO_2 NP with a diameter of 270 nm, detailed in Figure S5. This comparison reveals that, in contrast to the insulating state, the ED and MD contributions closely align, leading to diminished backscattering.

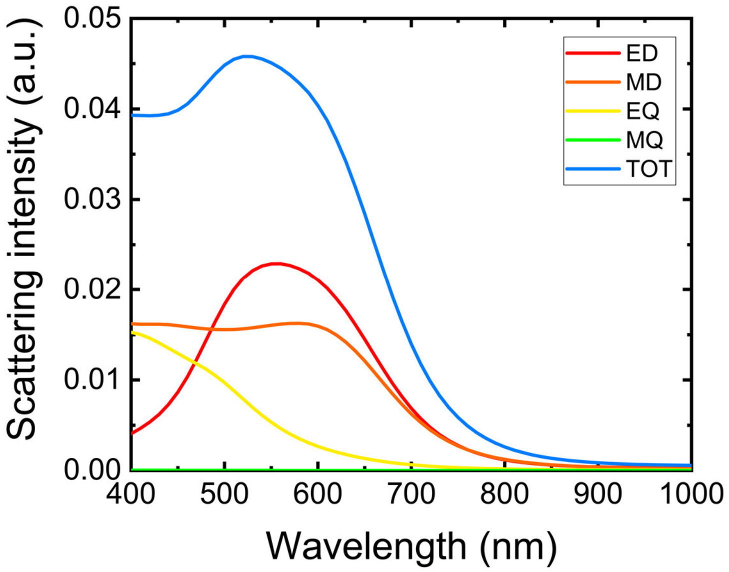

Figure S5: Decomposition of the total scattering spectra simulated for the metallic state VO_2 NP with $d = 270$ nm.

To demonstrate that BS strategies with quasi-first Kerker's wavelength can achieve better optical modulation effects in the visible light region, we have measured VO_2 NPs of various diameters for both FS and BS set up, with the results shown in Figure S6.

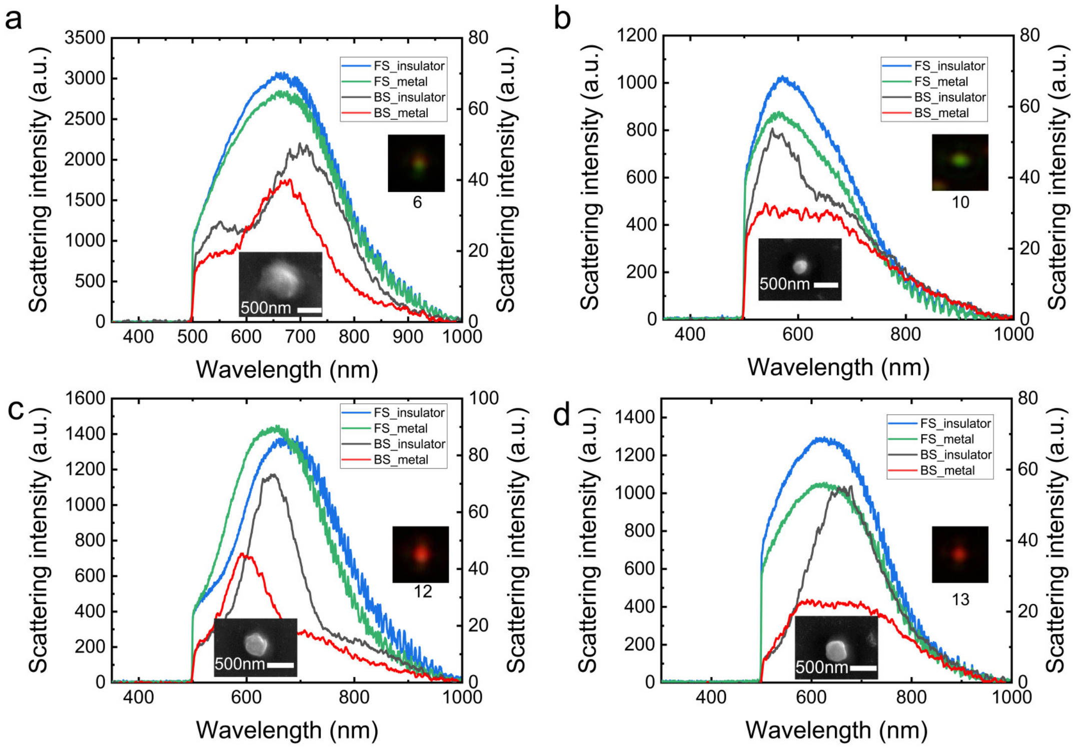

Figure S6: The measured scattering spectrum of VO_2 particles with a diameter of $d = 548$ nm, 284 nm, 412 nm, 380 nm, which confirms the BS strategy has a high modulation depth in the visible region.

In Figure S7, we present the *x-z* plane two-dimensional radiation patterns of the VO_2 NP calculated at 835 nm for the metallic and insulating phases, which correspond to a strong BS with quasi-second Kerker's wavelength. The constructive interference between the ED and MD modes in the backward direction and the destructive interference in the forward direction are responsible for the highly directional scattering observed.

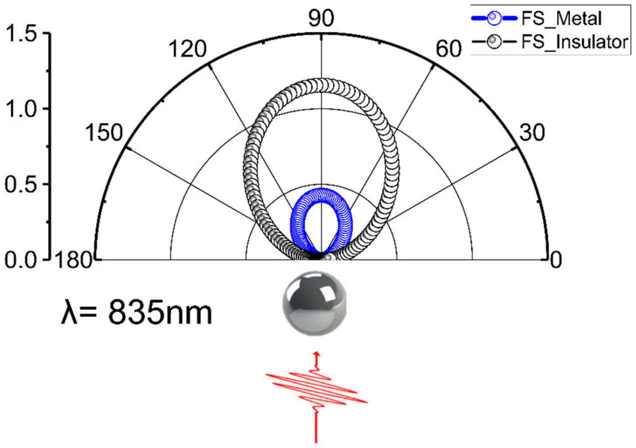

Figure S7: The FS scattering patterns of VO_2 NP with a radius $d = 240$ nm calculated at wavelengths of $\lambda = 835$ nm.

We have performed experiments to confirm that the scattering spectrum of VO_2 NPs can be modulate by heating platform without lasers. Figure S8 shows the evolution of BS spectrum with increasing temperature for the VO_2 NP with a $d = 240$ nm. It is shows that when the temperature reaches 341K, the BS spectrum of VO_2 NP decrease significantly, same as laser induced metal insulator transition process. Note that since the heating platform is opaque, we can only measure BS spectrum of VO_2 NPs. The dependence of temperature at the peak(~640 nm) of the BS spectrum on the temperature is shown in Figure S8 (b), which unambiguously shows the directional scattering of VO_2 NPs can be modulation using heating platform.

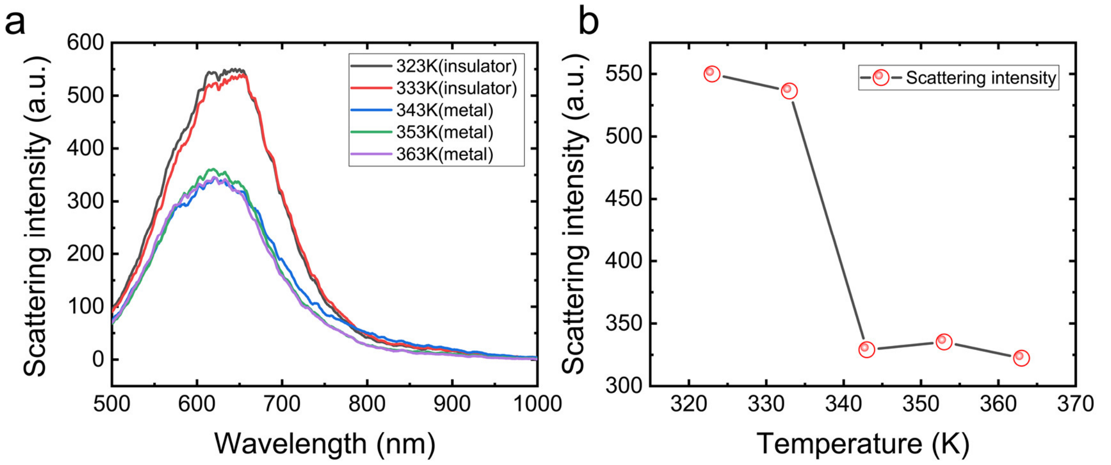

Figure S8. (a) Measured BS spectra of the VO_2 NP with increasing temperatures induced by heating platform. (b) The dependence of the intensity of the BS spectrum at the peak (~640 nm) on the temperature.

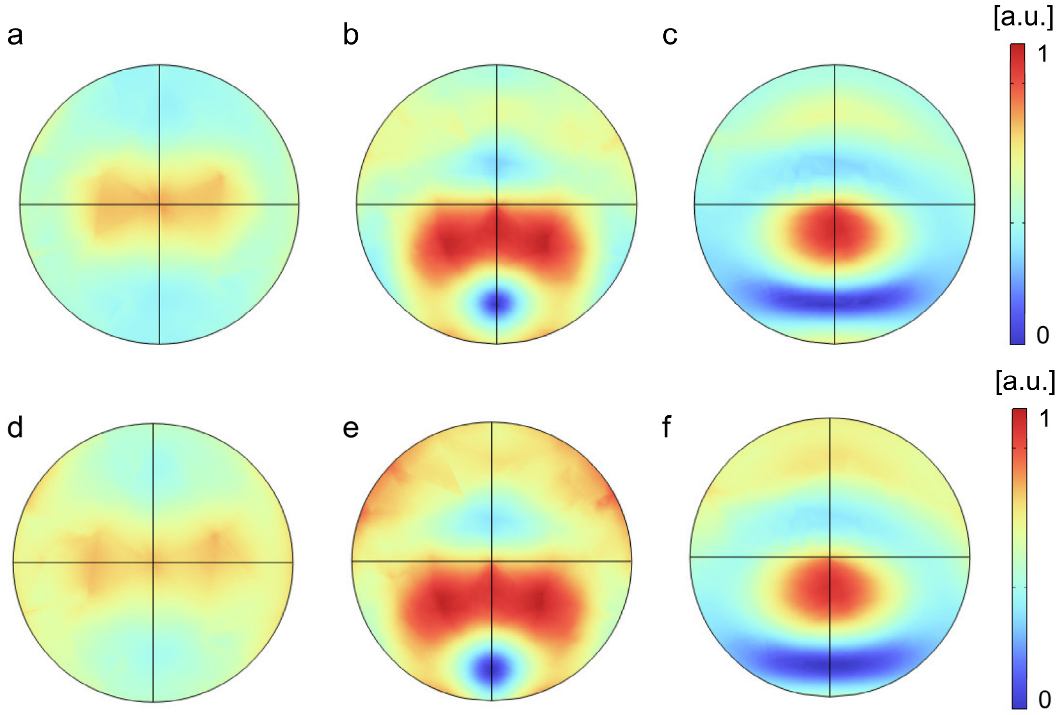

Figure S9: Electric field distribution of insulating (a-c) and metallic (d-f) state VO_2 NPs calculated for a diameter (*d*) of 270 nm with a wavelength 640 nm. Here, the a, b and c represent *xy*, *yz* and *yz* plane, respectively. The incident beam propagates along the z-axis with a x-direction polarized.

Ref.	Materials	Wavelength	Modulation depth	Modulation scheme	Modulation frequency/ Switching times
Dang Yuan Lei et al. [1]	VO_2+Au colloid	630nm	31%	Thermally	No/No
Dang Yuan Lei et al. [2]	VO_2+Au antenna	930nm	73%	Thermally	No/No
Otto L Muskenset al.[3]	VO_2 antenna	950-1500nm	39%	Photo-thermal	0.2MHz/2×10^6
Nikita A. Butakov, et al.[4]	VO_2 disk array	1500nm	14%	Thermally	No/No
Mohammed Reza M. Hashemi et al. [5]	VO_2, SiO_2, Au antenna	3mm	52%	Electrically	No/No
This work	VO_2 sphere	640 nm	56%	Photo-thermal	50Hz/5×10^4

Table 1. Summary of previously experimental demonstration of VO_2 based optical modulator.

[1] Lei, D. Y.; Appavoo, K.; Sonnefraud, Y.; Haglund Jr, R. F.; Maier, S. A. Single-particle plasmon resonance spectroscopy of phase transition in vanadium dioxide. Optics letters 2010, 35, 3988-3990.

[2] Lei, D. Y.; Appavoo, K.; Ligmajer, F.; Sonnefraud, Y.; Haglund Jr, R. F.; Maier, S. A.Optically-triggered nanoscale memory effect in a hybrid plasmonic-phase changing nanostructure. ACS Photonics 2015, 2, 1306-1313.

[3] Muskens, O. L.; Bergamini, L.; Wang, Y.; Gaskell, J. M.; Zabala, N.; De Groot, C.; Sheel, D. W.; Aizpurua, J. Antenna-assisted picosecond control of nanoscale phase transition in vanadium dioxide. Light: Science & Applications 2016, 5, e16173-e16173.

[4] Butakov, N. A.; Valmianski, I.; Lewi, T.; Urban, C.; Ren, Z.; Mikhailovsky, A. A.; Wilson, S. D.; Schuller, I. K.; Schuller, J. A. Switchable plasmonic-dielectric resonators with metal-insulator transitions. Acs Photonics 2018, 5, 371-377.

[5] Hashemi, M. R. M.; Yang, S.-H.; Wang, T.; Sepúlveda, N.; Jarrahi, M. Electronicallycontrolled beam-steering through vanadium dioxide metasurfaces. Scientific Reports 2016, 6, 35439.
